# Health information generation and utilization for informed decision-making in equitable health service management: The case of Kenya Partnership for Health program

**DOI:** 10.1186/1475-9276-4-8

**Published:** 2005-06-24

**Authors:** Nzioka M Solomon

**Affiliations:** 1 HSV-HIV project, Moi University-Indiana University Partnership Program; Faculty of Health Sciences, Moi University, Eldoret-Kenya

**Keywords:** built-in health information management, communities' health needs, service-linked data, social, cultural, political and economic determinants of health, community-based health information management, community-based health surveillance, health seeking behaviour, Healthy Villages Initiative

## Abstract

**Context:**

**The **Kenya Partnership for Health (KPH) program began in 1999, and is currently one of the 12 field projects participating in the WHO's 'Towards Unity for Health initiative' implemented to develop partnership synergies in support of the Primary Health Care (PHC) approach [1].

**Content:**

This paper illustrates how **P**rogram-linked **I**nformation **M**anagement by **I**ntegrative-participatory **R**esearch **A**pproach (PIMIRA) as practised under KPH has been implemented within Trans-Nzoia District, Kenya to enhance community-based health initiatives. It shows how this model is strategically being scaled-up from one community to another in the management of political, social, cultural and economic determinants (barriers and enhancers) of health.

**Objective:**

Target rural communities in the development of a community-based health information management and feedback initiatives that can provide insights on the social, cultural, political and economic determinants of health for utilization in informed health service management.

**Key Findings and Achievements:**

1. Cues for health seeking and health service utilization are determined by the social, cultural, political and economic factors as seen by the individual and as defined by the community but not due to the pathological nature of the illness.

2. Establishment of community-based health surveillance and health action initiatives as the best practices in transferring health as a resource that can be 'owned and guarded' by the community.

3. Establishment of Healthy Villages Initiative (HVI) through which health service delivery and scale-up can be sustained at the community level.

4. Provision of actionable health information necessary for health planning and evaluation of preventive health programs thorough PIMIRA.

**Conclusion:**

It has been realized that for every one person who visits a health facility for medication, there are nine others who had the same condition but sought health care from other sources including self-medication and five others who never sought health care. Innovative means of involving the community in health information management and utilization such as PIMIRA are hence the best ways of guaranteeing equitable delivery of health services that are accessible and sustainable by the community.

## 1.0 Introduction

### 1.1 Setting of KPH program

The focus of this article is to present the success story of closing the information-utilization gap through community-based initiatives by specifically narrowing down to the Program-linked Information Management by Integrative-participatory Research Approach (PIMIRA) and its role in promotion of sustainable health service delivery through Healthy Villages Initiative (HVI) as applied in the Kenya Partnership for Health (KPH) program. KPH is one of the 12 world wide field projects participating in World Health Organization's Towards Unity for Health (TUFH) initiative being implemented in support of the PHC pillars; namely inter-sectoral collaboration, community involvement and use of appropriate technology in order to deliver sustainable health services [2]. The focus of KPH program is capacity building and social structural strengthening at the community level for health information management and utilization in decision making, by use of health facility based routine data on reported morbidity and community based health surveillance. KPH prototype field program is based in Trans-Nzoia District of Rift Valley Province within the Republic of Kenya. It is implemented in the whole district but for effective and efficient monitoring and progress reporting, KPH is comprehensively undertaken in Amuka Sub-location of Kaisagat located in Kwanza Division within Trans-Nzoia District. Trans-Nzoia district is at an attitude of 1800 M above sea level with a high water table coupled with an annual rainfall of 1200 MM and a mean temperature of 18.6°C.

### 1.1 Background of KPH Program

Moi-University Faculty of Health Sciences offers undergraduate degrees in. Environmental Health, and Nursing whereby a Community-Based Education and Service (COBES) approach is applied in the training programs. COBES sessions are undertaken in each year of study for a period of six continuous weeks whereby students are assigned to a field -based program for hands-on experience. During the second year of Environmental health studies, students may participate in an elective course for six weeks at their place of choice so as to enhance any special skills they may want to develop and utilize during their career. For my elective course, I was assigned to the AMREF Water and Sanitation (WATSAN) project based in Kitui district of Eastern province.

### 1.3 Problem Statement and Justification

A rapid appraisal of the WATSAN project was one of the key activities I undertook. The two broad areas I considered were the project's performance against pre-set targets and the influence of the project on the beneficiary community's health profile. To the satisfaction of the project implementers, there were definite achievements in the number of springs and shallow wells protected as well as the number of Ventilated Improved Pit (VIP) latrines constructed and replicated by the community. However there was uncertainty in gauging the community's health outcome due to unavailability of service-linked data that can be inferred on the course-effect measurement scale. There was an intrinsic need to share and collaborate data from varied sources; not only from the routine health facility records which were inconsistently maintained but also incomplete. The records did not explain the community's health seeking behaviour and health service utilization. This gap between health information management and utilization formed the bulk of the work that I have been undertaking in search of a solution that can bridge the gap.

## 2.0 Conceptual framework

### 2.1 Empirical Argument from Literature Review

From the health information management and decision-making gap identified under the Kitui WATSAN project, I undertook review of approaches that could be applied to delineate the existing gap. From review, it was established that unfortunately this gap also contributes greatly to the utilization or non-utilization of health services due to the social, cultural, political and economic determinants of health that could not be captured from the routine health records. Theo Lipperveld, in his article on Health Information System, suggests that no single data source can provide all of the information required for planning and management of health services [3]. We sought an approach that would promote health information generation and utilization at the community level to solve the intricacies of health disparities due to differences in social, cultural, political and economic determinants of health. Insight was provided by operations research as well as community participatory training approaches that have been developed. However, a critical review of these broad areas of operations research and participatory community training approaches could not provide a solution to the prior identified problem. A search for a model that could not only combine the two broad approaches but also provide a health information-action link from the health services was undertaken.

Such community-based initiatives reviewed and applied over the five years in search of a model that could be applied to close the gap between health information generation, processing, dissemination and utilization of service-linked health information included development and utilization of community-based training toolkit such as in Participatory Hygiene and Transformation Training (PHAST), Child-to-Child and Participatory Rural Appraisal (PRA) approaches as well as a UNDP land planning approach referred to as Mèthode Appliècaution de le Planification et de Resèarchè (MAPIR). Review also covered health promotion models such as the PRECEDE-PROCEED model, the Health Education/Promotion model from Mark et al (1992), and the 21^st ^Century Field model on determinants of Health as adapted in Robert G. et al [4]. The section below gives an outline of the provisions of 21^st ^Century Field model on determinants of Health.

#### 2.1.1 The '21st-Century Field Model'

**Figure 1:**
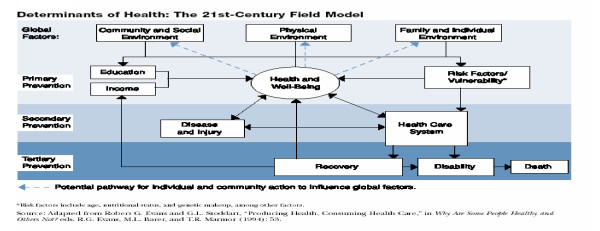
Determinants of Health: The 21^st ^Century model.

In the five year review, the 21^st ^field model was found to be the most comprehensive model that considers determinants of health in its broadest sense. It is unlike the modern practice of pathological orientation in which unfortunately the outcomes of preventive programs are also oriented to in the exclusion of social, cultural, political and economic determinants of health and well-being (see figure 1: Determinants of Health: The 21^st ^Century model).

#### 2.1.2 'The 21st-Century Field Model' Explanation

According to the 21^st ^century field model on determinants of health, there are three broad levels on which health can be promoted; primary, secondary, and tertiary. The environment variables that should be considered relate to primary prevention ("how do we keep ourselves well?"), secondary prevention ("if we are getting sick, how can we detect these conditions early?"), and finally tertiary prevention ("if we are sick, how do we get the best care?"). Secondary prevention involves activating the health care system to reduce the prevalence of disease and injury which can lead to recovery, disability, or death, or disease and injury can be resolved outside the health care system with appropriate self-care and good decision making.

According to the 'the 21st-century field model', recovery from an illness or injury can return an individual to productive pursuits, which feed back to the global factors at the top of the model. The big question being answered in this model is how the world can attain better health in the 21st century? Then as the model demonstrates, there are many paths of influence, each presenting its own challenges for the future. The components of the model that offer opportunities for improvement through communications and public health intervention are the community/social, Physical and family/individual environments. WHO has estimated that a poor physical environment is responsible for about one-fourth of all preventable diseases. For example, tropical regions are ideal environments for the transmission of many diseases including malaria, schistosomiasis, and diarrhea. Environmental conditions account for an estimated 90 percent of health problems caused by malaria. Environmental threats to human health can be divided into "traditional hazards" associated with a low level of economic development, and "modern hazards" associated with industrialization. Traditional hazards related to poverty and low levels of economic development and include lack of access to safe drinking water, inadequate sanitation in the household. In the community these include indoor air pollution and inadequate solid waste disposal.

With the structural framework outlined in the 21^st ^Century Field Model, PIMIRA takes a further step of addressing the determinants of health at the community level by providing service-linked information for the purpose of enhancing ownership to health as a resource that can be 'cherished and guarded' by the community.

#### 2.1.3 Birth of Program-linked Information Management by Integrative-participatory Research Approach (PIMIRA) as the Information-Utilization Magic Bullet

This article presents the resultant model developed when none of the reviewed models could respond to the initially identified gap under the Kitui WATSAN project; namely that of health information and community participation in health service delivery. The resultant model described hereby is referred to as **P**rogram-linked **I**nformation **M**anagement by **I**ntegrative-participatory **R**esearch **A**pproach (PIMIRA), which has been developed since 1999. PIMIRA is currently being implemented hand-in-hand with other community advocacy-for-health-action approaches such as the polythene free initiative, Healthy Villages Initiative and the annual Health festivals extravaganza; it is the bond that is transforming the community advocacy-for-health-action approaches to health ownership by the community in the context of 'our health-our good-our responsibility'.

The PIMIRA model has proven to be effective in closing the gap between health information generation, processing, dissemination and utilization by forming the soft bond of providing health information relevant for informed decision-making to the community, health managers, policy makers, health professionals and health training institutions.

#### 2.1.4 PIMIRA Principles

##### Assumptions in PIMIRA

i. Those who create decisions will be committed to following them through sustainable health development

ii. Communities are capable of accurately describing their present situation/ problems and visualizing possible improvements

##### Underlying Principles

i. People have beliefs about the causes of diseases which may or may not be consistent with the scientific explanations of the disease

ii. No lasting change in people's behavior may occur without health awareness, understanding and believing in the change

##### PIMIRA Operational Principles

i. The best way to achieve sustainable health improvements is to take an incremental approach, starting with what the community has and building upon a series of changes

ii. Self-esteem and associative strength is a prerequisite to decision-making and follow-up.

iii. Local people should be the health analysts and presenters of their own health situation while health workers play the role of catalytic facilitation.

iv. Any innovative initiative should apply adaptable participatory learning process (creative) rather than lay down a prescriptive set of tools to be followed.

v. Community Health training by health information visualization reduces marginalization and promotes equitable health access

vi. Multi-disciplinarity and diversification of information sources, tools and techniques avoids uni-sectorial approaches to reality by enabling analysis from varied outcome determinants

#### 2.1.5 Community Based Toolkit (for Operations Research)

i. Pocket Chart (PC) – an investigative and evaluative tool used to collect and tabulate data on where people defecate, where they collect water and their information and communication structure.

ii. Community mapping – showing the available water supply resources, permanent mosquito breeding sites and pillar stones in information and communication within the community as well as disease distribution by lay definitions.

iii. Resource maps – showing the income generating activities the community is involved in.

iv. Flow charts – showing the possible water and food contamination routes as well as the existing information flow structure.

v. Matrix classifications – a set of pictures of common causes and barriers of health and communication. They utilize epidemiological principles to pass health concepts to the community by way of visualization.

vi. Venn diagrams – Collects information about the traditional or modern organizations involved in the management of local water resources and information systems.

vii. Community surveillance tally cards – cards containing visual representations of the main signs of water related diseases and malaria vis-à-vis community routine activities like school attendance and provision of casual labour to the agricultural farms as is usually the case.

viii. Facility morbidity tally sheets – tabulation sheets for recording village specific water related diseases and malaria as they are reported in the community health facilities.

ix. Historical analytic charts and Seasonal calendars amongst others documents how the community have been handling certain diseases and the traditional seasonal calendar scheduling of activities from the community historical perspective.

## 3.0 KPH Interventions/Preventive Services

Hardware of KPH program includes provision of Insecticide Treated Mosquito nets (ITNs), indoor residual spraying against mosquitoes, safe water supplies and promotion of latrine ownership while the software (means to sustainable social and economic development) includes capacity building and social structural strengthening through community training, enhancement of Income Generating Activities (IGAs) through micro-enterprising, enhanced community involvement in health information surveillance through the operations research (PIMIRA) and finally stakeholder networking through the service-linked information dissemination.

### 3.1 The Big-4 Questions answered by PIMIRA

i. Can community-based health surveillance on the cultural, social, political and economic factors be utilized in addressing the determinants of health as well as enhancing management of health resources at community level?

ii. Can community involvement in health information management improve access, utilization and provision of equitable health services?

iii. What methods can be applied to promote direct involvement of the community in Health Information Management?

iv. Can community involvement in Health Information Management be utilized for effective scaling-up of health gains within the community?

### 3.2 The PIMIRA Structural Framework

**Figure 2:**
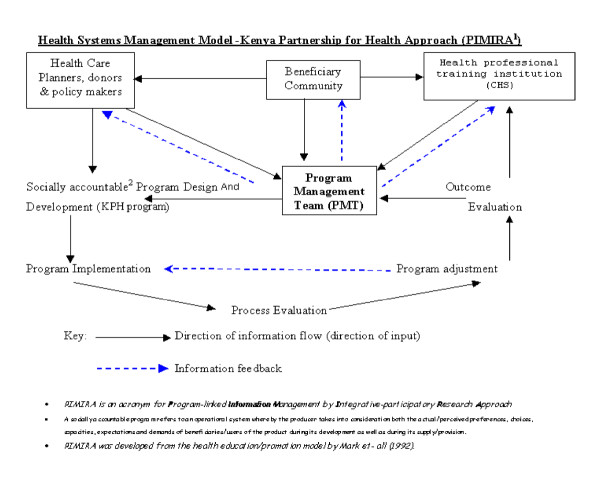
Health Systems Management Model: Kenya Partnership for Health Approach.

Program-linked Information Management by Integrative-participatory Research Approach (PIMIRA) answers the over- arching question of 'How do we provide service-linked information to different stakeholders in health care for informed decision-making and for delivery of sustainable health services from the beneficiary community's perspective? Answer to this question is illustrated in Figure 2: Health Systems Management Model: Kenya Partnership for Health Approach

#### Operations Research model: 'PIMIRA' Explanation

The model above represents the framework of a socially accountable health delivery system as projected under the KPH program. In such a system, information management is the power and the strength through which decision and actions regarding the preventive services being offered are monitored, evaluated and improved. The program management team is the conceptual and operational group targeted with developing or delivering socially accountable services answerable to beneficially community health needs. The team is basically a network of a multi-disciplinary oriented working group who oversee all the implementation, monitoring and evaluation of the program with controls or indicators for all social, cultural, political and economic determinants of health. It is an epicenter for centralized service-linked information management and dissemination for timely feedback on the program from all the concerned partners. The input/incoming arrows from the boxed texts to the PMT signify the basic information sources that should be critically sought in the initial design of a socially responsive (cost-effective, high quality, relevant and equitable) health delivery program.

The out-going box from the PMT signifies the concretized input from the other partners while the dotted blue lines represent the levels of action-linked feedback.

#### 3.2.2 A Critique of PIMIRA Model Suitability

Health information is identified as the key to successful partnership and sustainable health service delivery as well as the tool through which community involvement for ownership in health services can be based. PIMIRA was developed from the health education/promotion model by Mark et all (1992). The major deviations (to address weaknesses in Mark's model) are:-

1. Marks' model exclusively focused on health promotion schemes while PIMIRA model focuses on Human Resource for Health (HRH) development by use of community-based tool-kit.

2. Unlike the two feedback levels to project implementation and Project objectives recognized in Marks' model, PIMIRA considers the two and refocuses on the overall health service planners and the community as key decision-making levels for improvement of any health service.

3. PIMIRA model strongly links the health service planners, the community and the training institutions by way of mutually dependent information sources for common/shared health action

4. It puts targeted population into active partnership from the inception to the end of the sponsored health service project through the design, development and use of community-based toolkit.

5. Beneficiary community is the main actor in process evaluation. They continuously gather activity-based health attribute data, develop community health profile and subsequently decide on the cause of action for their own health.

6. Activity specific-health indicators and the community health profile (Objectively Verifiable Indicators) forms the basis for evidence-based health impact and outcome evaluation.

### 3.3 The Healthy Villages Initiative (Ripples analogy)

**Figure 3:**
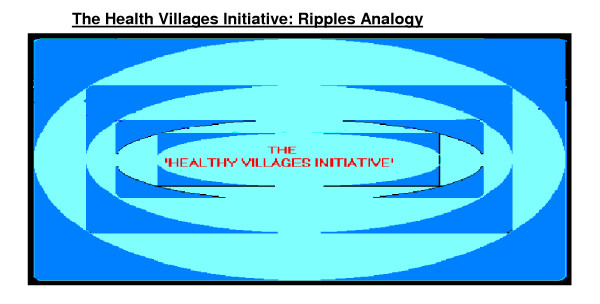
The Healthy Villages: Ripples Analogy.

This is the default method through which villages have embraced preventive services provided through PIMIRA as 'our health, our responsibility' and hence duplication of the health interventions in the villages through the neighborhood concept of development. This has been realized through public health field days being held within the healthy villages as illustrated in Figure 3: The Healthy Villages: Ripples Analogy.

A healthy village is defined as an administrative village in which minimum public health requirements to prevent malaria and diarrhea diseases have been undertaken and there has been demonstrable evidence on the latter. In future this will also include HIV/AIDS and Child Health Essential Services (CHES) a package that will include promotion of safe delivery practices, immunization, nutrition and prevention of childhood illnesses as outlined in 2003 UNDP report [5] and by Scott et al [6].

Public health field days are community awareness campaign/show days during which the neighbouring communities/villages are invited for education and awareness on best-integrated preventive practices against malaria (including Intermitted Pregnant Treatment -IPT) and diarrhea diseases. In future this will also include HIV/AIDS and CHES. The following session demonstrates what timely health information can contribute to enhance access and equity of health care services in relation to malaria, diarrhoel diseases and HIV/AIDS. This concept is based on the Ottawa Charter [7] and the 1998 Human Development report [8].

## 4.0 Achievements through PIMIRA community-based initiatives

Table 1 presents evidence of sustainable health service delivery by closing service-based information-utilization gap. It shows that though malaria had low fatality rate, it is the major contributor to individual financial resource drains in the community directly and indirectly since as collaborated from the community-based health surveys (conducted at base line), many people do not go to look for casual jobs as main contributor to children absenteeism at schools. These factors have direct major impact to the Trans-Nzoia community economic welfare were more than 75% of the natives are in the informal employment sector. Malaria morbidity was seen to directly be leading to reduced per capita earning and hence low purchasing power which narrows down to determination of where, when and how to seek medication. The community based surveillance toolkit implemented under PIMIRA led to the realization of the community's economic potential that can be achieved if malaria morbidity can be reduced and embracement of preventing mosquito breeding and bites within the Health Villages Initiative and hence realization of "Our Health, Our Responsibility".

**Table 1:** Evidence of sustainable health service delivery by closing service-based information-utilization gap *(Malaria as the main economic depressant from the community's perspective)*

***Top three diseases and their case fatality rate for the year 2001 (cue for community action)*.**				
**Disease**	**No. Of cases reported**	**Relative % Mobidity**	**No. Dead**	**Case fatality rate**

Malaria	18220	77%	180	0.9*
Pneumonia	1836	8%	128	6.9
Diarrhea diseases	3464	15%	80	2.3

Within the Health Villages, School Health clubs, market committees and Village Health Committees are now having shared health objectives in eliminating mosquito-breeding sites. Through public health field days, this has led to the neighboring villages demanding that the same preventive measures be undertaken in their villages. For the first time in many years, a malaria outbreak was not reported in Trans-Nzoia District since 2001 [9]. Through this, the roll back malaria initiative has got roots and is now rolling out from one village to the next and eventually we hope it will spread to the whole district [1].

**Figure 4:**
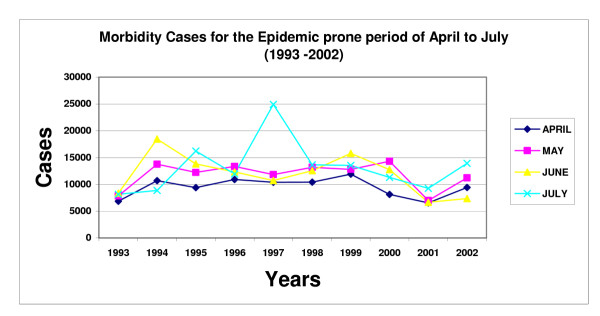
Morbidity Cases for the Epidemic prone period of April to July (1993 – 2002).

Related community actions includes social marketing of insecticide treated nets at a subsidized cost from population Service International (PSI), community mapping of mosquito breeding sites and their management, indoor residual spraying and house screening. Achievement of all these measures to 75% coverage has been our mark for a Healthy Village before a public health field day is held. A total of 9 public health field days have since been held and this is an indicator of coverage in the efforts of rolling back malaria in the community and hence increasing their economic productivity. With a district malaria epidemic threshold of 15,000 cases per month, the trend has been as presented in figure 4.

### 4.2 The case of Diarrhoeal Diseases Management as a cue for community action

**Figure 5:**
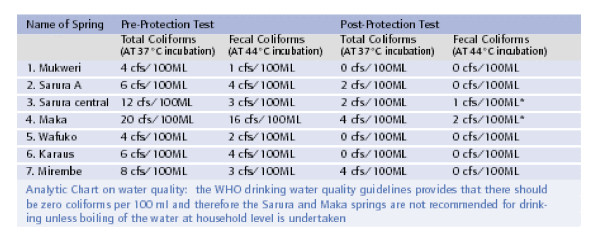
Testing outcome on Coli-forms in water springs before and after protection.

The reference community uses communal springs as their main sources of drinking water. To enhance community participation, spring committees were selected by the community to support the Village Health Committees in the coordination of spring protection activities such as collection of the community share of contribution. From the program side, this group was utilized for health communication to the community regarding safe water handling even at household level to prevent recontamination. The communal springs were tested for coli-forms before and after protection and test outcome is as presented in figure 5.

Using Moi University, Faculty of Health Sciences mobile paqua lab, the spring protection committee members where utilized in drawing both pre and post protection water samples and were present during incubation to test the levels of contamination. Having obtained the above results, the community Health Workers (CHWs) continued to administer the community-based surveillance tools after which the results was compared to the dispensary records on diarrhoeal diseases. Through subsequent community mapping charts, the community members were amazed to note the distinct changing patterns of diarrhoeal cases reported. Using the protected water sources as reference points to convince the community on the significance of water source protection in the reduction of diarrhoeal cases, more springs are now been protected. The community is now competing to contribute their share and hence demanding that the program contributes its share for their water sources to be protected. Unlike in the past where springs maintenance was the work of the Government or the NGOs who had sponsored their protection, the total number of 9 springs protected under the KPH program are maintained/repaired by the community.

The story of fecal contamination, its subsequent elimination by water source protection and the subsequent reduction of diarrhoeal diseases is taught every time there is a public health field day. This has dramatically increased the demand from the community for protected water sources. They are writing proposals and organizing themselves into spring committees without a significant health professionals' involvement.

All the community-advocacy for action activities are undertaken through self-help groups for which seed funds are given in the form of Insecticide treated mosquito nets as an income generating activity. Scaling up of the Health benefits from one village to another is through public health field days in which neighbouring villages are invited to see, touch and feel public health interventions within the Healthy Villages. This is the concept outlined in the 2003 WHO report [10].

### 4.3 HIV status as an indicator of testing facilities utilization

Table 2 presents evidence on how closing service-based information-utilization gap can led to sustainable health service delivery. Health facility utilization indicator is inversely proportional to the number of people testing positive out of those who turn up for actual testing. When the percentage indicator is high, most of the people who turn up for testing are actually positive and hence on inference, people who have had strong reasons to believe that they are infected are the only ones utilizing the testing facility. On the other hand, when the percentage indicator is low, it shows that most of the people who turn up for testing are end up being negative and hence on inference people who are utilizing the service have had other reasons or cues to action other than strong reasons to believe that they are infected. These other cues for health action may include and not limited to increased individual/societal health awareness on the need for the service on offer which for the case of HIV/AIDS in Trans-Nzoia District may be directly attributed to the community-based awareness strategies in place such as peer group counseling de-stigmatization campaigns through Village Health Committees, Market committees and School Health Clubs.

**Table 2:** HIV status as an indicator of utilization of testing facilities (Closing service-based information-utilization gap sustainable health service delivery)

**YEAR/ MONTH**	**1999**		**2000**		**2001**	
	
	**No. tested**	**% positive**	**No. tested**	**% positive**	**No. tested**	**% positive**
JAN	57	44	15	27	73	29
FEB	94	48	75	52	62	45
MAR	71	37	95	42	119	46
APRIL	55	55	71	45	27	37
MAY	56	45	88	45	139	44
JUNE	47	53	86	29	116	44
JUL	52	35	70	50	115	28
AUG	74	31	75	56	63	37
SEPT	57	54	109	45	68	44
OCT	64	53	41	29	124	29
NOV	67	39	101	54	104	34
DEC	64	58	47	15	92	26
TOTAL	758	552	873	489	1102	443
**Service utilization Indicator (%positive/%tested)**		73%		56%		40%

Looking at it from another angle, even when the cues for action are taken to be constant, then the utilization indicator in the case of HIV/AIDS shows that there is a progressive annual increase in the number of people seeking testing services at an early stage of infection.

As an evidence of information for decision-making loop completed from the above reporting, the year 2002 witnessed an affirmative action by the District Medical Officer of Health (DMOH) and the District Commissioner (DC) to the effect that data on all HIV/AIDS testing and related services kept by the different NGOs, private and mission organizations within the district should be centralized for analysis on regular intervals of every three months. This was in addition to the establishment and acceptance of the KPH program to operate in the District so as to coordinate data management and utilization for health action at all levels of care within the district.

## 5.0 Conclusions, recommendations and way-forward

The PIMIRA method has operationalized the 21^st ^Century Field model on determinants of health by addressing the social, cultural, political and economic determinants of ill health as defined in its outline on different environments affecting health and well-being. This is as contained in the PIMIRA operational principles outlined in this document.

Scaling up of this approach to include any new health package or cover a larger area is intrinsically provided for in PIMIRA through its congruent Health Villages initiative. KPH is hence a good case example of promoting health equity and access to health services by closing the information-utilization gap through community-based initiatives.

Recommendation is for logistical and financial resources to be availed for extension of the KPH coverage especially for community-based trainings and seed funds for income generating activities. The success PIMIRA has guaranteed the way forward for sustainable delivery of health services that are based on community's needs (social, political, economic and cultural) and hence establishment of socially responsive health programs.

The way-forward is to scale-up the Healthy villages initiative by including HIV/AIDS support programs for the People Living With AIDs (PLWAs) and People Affected by HIV/AIDs (PAHAs) as well as targeted interventions on Child Health Essential Services (CHES), a package that will include promotion of safe delivery practices, immunization, nutrition and prevention of childhood illnesses.

Finally as noted in the World Health report 2003, with a renewed commitment to the principles of PHC through strengthened health systems, real Millenium Development Goals (MDGs) and other national health priorities can be achieved. Shaping the future looks at the impact of the different threats to health and offers innovative and far-reaching solutions that will shape a better, healthier future for all – but only if the international community act now.
